# Stratified randomisation: a hidden form of clustering?

**DOI:** 10.1186/1745-6215-12-S1-A22

**Published:** 2011-12-13

**Authors:** Brennan C Kahan, Tim P Morris

**Affiliations:** 1 MRC Clinical Trials Unit, 125 Kingsway, London, WC2B 6NH, UK

## Objectives

Many randomised trials use stratified permuted blocks or minimisation to balance key prognostic variables between treatment groups. It is widely argued in the statistical literature that any balancing variables should be adjusted for in the analysis, however a review of major medical journals shows that this is not commonly done. Our objective was to determine the effects of an unadjusted analysis after balancing.

## Methods

The statistical properties of an unadjusted analysis after balancing are explored using theoretical results. A major simulation study using data from 5 trials is performed to determine the potential impact in real life situations.

## Results

We show that balancing on baseline covariates leads to correlation between the treatment groups (similarly, cluster randomised trials lead to correlation within treatment groups). If this correlation is ignored, and an unadjusted analysis is performed, the estimated variance of the treatment effect will be biased upwards, resulting in type I error rates that are too low, and a reduction in power. Conversely, an adjusted analysis results in nominal type I error rates, and optimal power.

## Conclusions

Prognostic variables that have been balanced between treatment groups in the randomisation process should be adjusted for in the analysis. Unadjusted analyses lead to invalid results, whereas adjusted analyses maintain nominal properties.

